# Differential Effects of the Toll-Like Receptor 2 Agonists, PGN and Pam3CSK4 on Anti-IgE Induced Human Mast Cell Activation

**DOI:** 10.1371/journal.pone.0112989

**Published:** 2014-11-14

**Authors:** Yangyang Yu, Kwok Ho Yip, Issan Yee San Tam, Sze Wing Sam, Chun Wai Ng, Wei Zhang, Hang Yung Alaster Lau

**Affiliations:** 1 Shenzhen Key Laboratory for Translational Medicine of Dermatology, Biomedical Research Institute, Shenzhen Peking University-The Hong Kong University of Science and Technology Medical Center, Shenzhen, Guangdong Province, China; 2 School of Biomedical Sciences, Faculty of Medicine, The Chinese University of Hong Kong, Hong Kong, SAR, China; University of London, St George's, United Kingdom

## Abstract

Mast cells are pivotal in the pathogenesis of allergy and inflammation. In addition to the classical IgE-dependent mechanism involving crosslinking of the high-affinity receptor for IgE (FcεRI), mast cells are also activated by Toll-like receptors (TLRs) which are at the center of innate immunity. In this study, we demonstrated that the response of LAD2 cells (a human mast cell line) to anti-IgE was altered in the presence of the TLR2 agonists peptidoglycan (PGN) and tripalmitoyl-S-glycero-Cys-(Lys)4 (Pam3CSK4). Pretreatment of PGN and Pam3CSK4 inhibited anti-IgE induced calcium mobilization and degranulation without down-regulation of FcεRI expression. Pam3CSK4 but not PGN acted in synergy with anti-IgE for IL-8 release when the TLR2 agonist was added simultaneously with anti-IgE. Studies with inhibitors of key enzymes implicated in mast cell signaling revealed that the synergistic release of IL-8 induced by Pam3CSK4 and anti-IgE involved ERK and calcineurin signaling cascades. The differential modulations of anti-IgE induced mast cell activation by PGN and Pam3CSK4 suggest that dimerization of TLR2 with TLR1 or TLR6 produced different modulating actions on FcεRI mediated human mast cell activation.

## Introduction

Mast cells which express high-affinity IgE receptor (FcεRI) are key immune effector cells in allergic associated diseases such as asthma and atopic dermatitis. In recent years, following the growing knowledge on the roles of mast cells in innate immunity, interaction between FcεRI -mediated allergic responses and Toll-like receptor (TLR)-mediated reactions has attracted more and more attention [1]–[3]. Epidemiological studies suggest that increased exposure to microbial compounds decreases the risk of developing allergies in children [4], [5]. However, the outcomes from TLR2 and FcεRI co-stimulation are controversial in studies using different in vitro models.

Several literatures described the relationship between these two systems and indicated the synergistic release of cytokines after co-stimulation of antigen with TLR2 ligands from mast cells [3], [6], [7]. Synergistic effect on cytokines production was contributed to the synergistic activation of MAPKs and their related transcriptional factors c-Jun, c-fos and ATF-2 [3]. In contrast, other groups have reported inhibitory effects on mast cell degranulation and cytokines production which were either through decreased FcεRI expression or suppression of calcium mobilization and Erk phosphorylation due to pre-treatment with different TLR2 ligands [1], [2], [8]. Most of these studies were carried out by investigating mouse mast cells. Only one study used the human mast cells line LAD2 cells and observed the suppression of FcεRI expression and antigen-induced mast cells degranulation upon TLR2 ligands pre-treatment [2]. However, the modulatory effects of TLR2 on FcεRI -induced release of de novo synthesized mediators from human mast cells are unclear.

Activation of TLR2 in mast cells induced the release of various inflammatory mediators [9]. Signaling pathways involved in TLR2-mediated activation initiates through recruitment of the adaptor molecules, myeloid differentiation protein 88 (MyD88) [3]. On the other hand, FcεRI activation of mast cells involves multivalent binding of antigens to IgE bound FcεRI on mast cells and the recruitment of immunoreceptor tyrosine activation motifs (ITAMs) [10]. However, similar usage of MAPKs, NF-κB, PI3K-Akt pathways and the involvement of calcium mobilization are shared by both of the activation systems [11]–[13].

In the following study, we investigated the modulatory effects of TLR2 ligands on human mast cell line LAD2 cells activation in response to anti-IgE in the context of degranulation and release of de novo synthesized mediator IL-8. Two widely accepted TLR2 ligands bacterial peptidoglycan (PGN, ligand for TLR2/TLR6) and tripalmitoyl-S-glycero-Cys-(Lys)4 (Pam3CSK4, ligand for TLR2/TLR1) were employed to investigate if there are differences between the effects of TLR2/TLR1 and TLR2/TLR6 heterodimers on human mast cell activation. Preliminary studies on signaling pathways involved in the events were further studied.

## Materials and Methods

### Human mast cell culture

The Laboratory of Allergic Disease 2 (LAD2) human mast cells were kindly provided by A. Kirshenbaum and D. Metcalfe (NIH, USA) [14]. Cells were maintained in StemPro-34 medium supplemented with 10 ml/l StemPro nutrient supplement, 1100 penicillin- streptomycin, 2 mmol/l L-glutamine, 100 ng/ml human stem cell factor, and 50 ng/ml interleukin-6 in an atmosphere containing 5% CO_2_ at 37°C. The culture medium was replaced every 2 weeks and the cells were kept at a density of 10^5^ cells/ml. Cells were incubated with 0.5 g/ml human myeloma IgE overnight prior to further treatment.

### Chemical Reagents

Peptidoglycan (PGN) from S. aureus, mouse anti-human immunoglobulin E antibody (ε-chain specific) (anti-IgE) were purchased from Sigma. Human Myeloma IgE was from Merck. Pam3CSK4 was purchased from Invivogen. SP600125, SB203580, PD98059, ciclosporin and Bay11-7821 were from Tocris. Wortmannin was from Cayman. FITC-conjugated anti-human FcεRI antibody and FITC-conjugated mouse IgG2b isotype control were purchased from eBioscience. When chemicals were dissolved in DMSO, the final concentration of DMSO did not alter the normal response of LAD2 cells.

### Flow cytometry assay

LAD2 cells (without sensitization with IgE) were incubated with 50 µg/mL PGN or 20 µg/mL Pam3CSK4 for 24 h. Cells were washed twice with PBS and blocked with human IgG in FACS buffer for 15 min on ice, and then washed and resuspended in FACS buffer. Cells were incubated with FITC-conjugated anti-human FcεRI antibody, FITC-conjugated mouse IgG2b isotype control or FACS buffer respectively (served as blank control) for one hour in dark on ice. Cells were then washed and transferred to round bottom FACS tube for analysis by using BD LSRFortessa cell analyzer.

### Degranulation assay

β-hexosaminidase (β-hex) is an enzyme contained in the cytoplasmic granules of mast cells and the degree of release of this enzyme into the supernatant provides an indication of the degranulation process following mast cell activation. LAD2 cells were respectively incubated with different stimuli for 30 min and β-hex release was measured. β-hex in supernatants and cell lysates was determined by use of a colorimetric assay in which release of p-nitrophenol from 4-nitrophenyl N-acetyl-β-D-glucosaminide was measured [1]. The absorbance was measured at 405 nm by using a multiplate reader. 605 nm reading was taken as a reference. The percentage of β-hex release was calculated as the percentage of the total β-hex content. All results were corrected for spontaneous β-hex release that was less than 5%.

### IL-8 measurement

LAD2 cells were pre-incubated with various inhibitors for the corresponding time periods prior to incubation with different stimuli for 24 h to allow synthesis and release of IL-8. The release of IL-8 in the supernatants was measured by ELISA assay (BD Biosciences) according to the manufacturer’s instructions. All results were corrected for spontaneous IL-8 release that was less than 22 pg/10^6^ cells.

### Intracellular Ca^2+^ mobilization assay

LAD2 cells were loaded with 2 µM Fura-2 AM (Invitrogen) for 30 min at 37°C. The cells were washed three times and then resuspended in HEPES buffer with human albumin prior to challenging with different stimuli. Fura-2-loaded mast cells were viewed with an Olympus inverted IX51 microscope. The images were captured with a CCD camera at every 10 s intervals. Fluorescence images were obtained at wavelengths of 340 and 380 nm with an emission wavelength of 510 nm. The fluorescence ratio of 340 to 380 nm was measured and the overall levels of calcium influx were compared with area under the curve analysis. F_1_/F_0_ was the fluorescence ratio of time point X divided by the ratio of time point zero.

### Statistical analysis

Statistical significance was determined by student *t*-test, one-way and two-way ANOVA. Differences were considered significant at a P value of less than 0.05. All data are expressed as means±SEMs.

## Results

### TLR2 ligands suppressed LAD2 cells degranulation induced by anti-IgE

**Figure 1:**
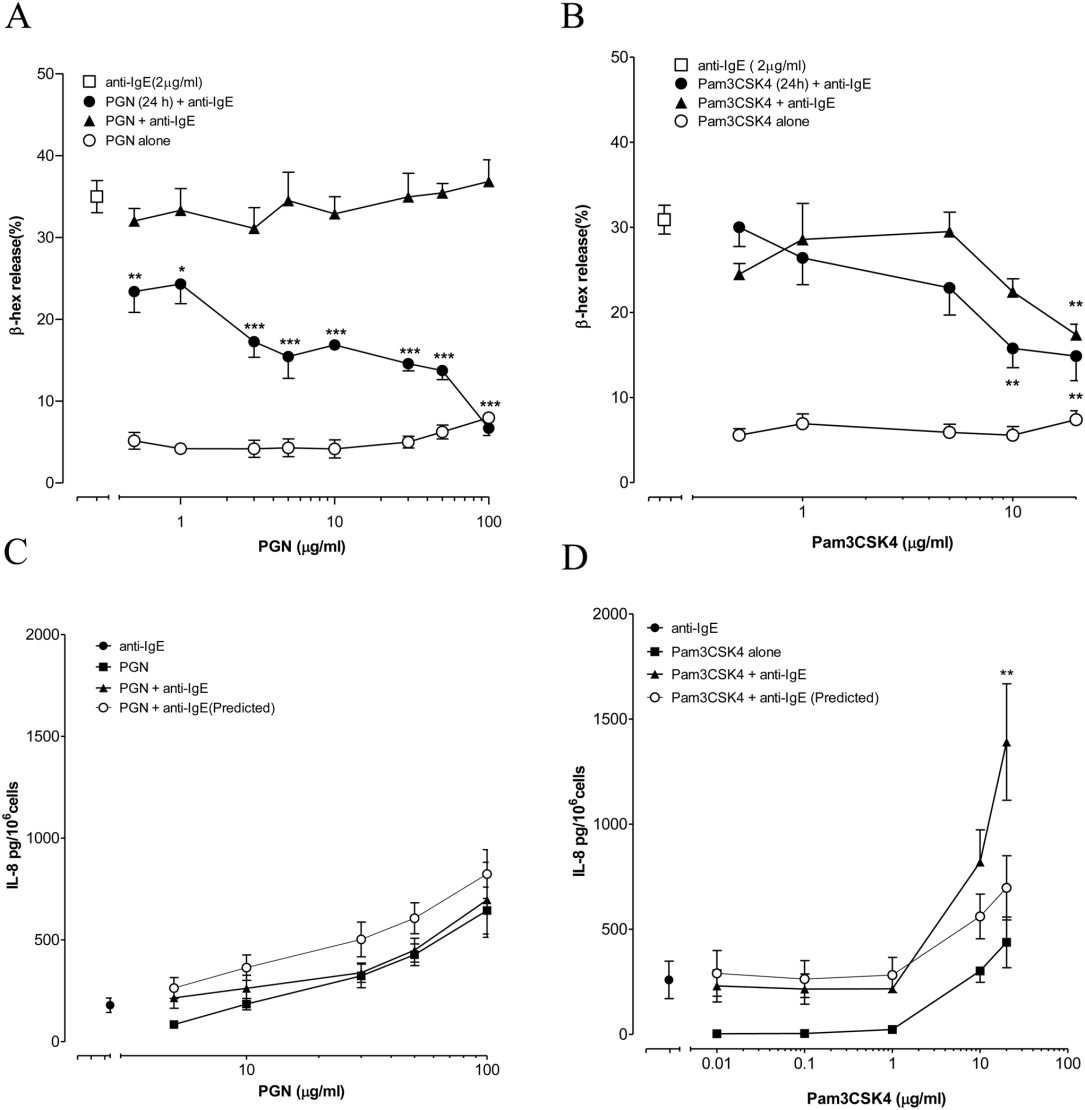
Effect of TLR2 ligands on anti-IgE induced degranulation and IL-8 release from LAD2 cells. (A, B) LAD2 cells were incubated with only PGN or Pam3CSK4 for 30 min (○). LAD2 cells were incubated with anti-IgE (2 µg/ml) at the same time (▴) or after 24 h pre-incubation (•) with PGN or Pam3CSK4. The levels of β-hex release induced by anti-IgE alone and in the present of TLR2 ligands were compared with one-way ANOVA and Dunnett’s multiple comparison tests. *p<0.05, **p<0.01, ***p<0.001 (n = 3–5). (C, D) LAD2 cells were incubated alone with anti-IgE (2 µg/ml, •), PGN/Pam3CSK4 (▪) or combination of anti-IgE with PGN/Pam3CSK4 (▴) for 24 h. Two-way ANOVA and Bonferroni posttests were applied to compare the actual amount of IL-8 released with the predicted value obtained by adding the individual amounts released by anti-IgE and PGN or Pam3CSK4 (○). **p<0.01 (n = 5).

PGN and Pam3CSK4 did not induce significant release of β-hex on their own (Fig. 1A, B) while anti-IgE induced a release of around 30% of total β-hex after a 30 min incubation with LAD2 cells. When added to LAD2 cells simultaneously with anti-IgE, PGN had no significant effect on anti-IgE induced β-hex release while Pam3CSK4 inhibited the release only at the highest concentration of 20 µg/ml tested. In contrast, dose-dependent inhibition of anti-IgE induced β-hex release from LAD2 cells was observed with both TLR2 ligands when LAD2 cells were activated after pre-incubation with the TLR2 ligands for 24 h. (Fig. 1A, B).

### TLR2 ligands differentially regulated anti-IgE induced IL-8 release from LAD2 cells

PGN and Pam3CSK4 induced IL-8 release from LAD2 cells dose dependently over 24 h incubation (Fig. 1C, D). Synergistic release of IL-8 was observed when Pam3CSK4 and anti-IgE were added simultaneously to LAD2 cells with maximum release of 1390.8±595.0 pg/10^6^ cells at 20 µg/ml of Pam3CSK4, which was over 200% higher than the additive combined release (Fig. 1D). In contrast, PGN did not demonstrate similar synergism and the actual release induced by simultaneous incubation with anti-IgE and PGN was not significantly different from the additive combined release (Fig. 1C).

### TLR2 ligands did not reduce the expression of FcεRI on LAD2 cells

**Figure 2:**
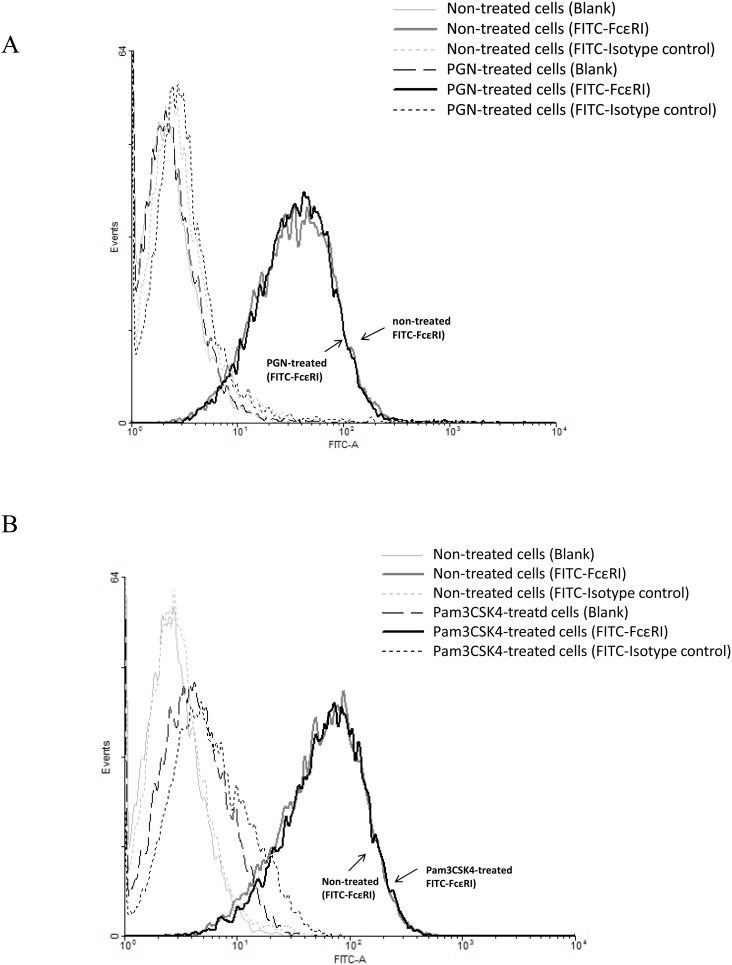
Effects of PGN and Pam3CSK4 on the expression of FcεRI on LAD2 cells. LAD2 cells (without IgE sensitization) were incubated with PGN (50 µg/ml) (A) and Pam3CSK4 (20 µg/ml) (B) for 24 h and FcεRI surface expression was analyzed by flow cytometry after cells were incubated with FITC-conjugated anti-human FcεRI antibody, FITC-conjugated mouse IgG2b isotype control or FACS buffer for specific labelling of FcεRI, isotype and blank control respectively. The FcεRI expression of cells that were not treated (grey curve) or treated (blank curve) with the TLR2 ligands was not different as shown. Results were representative of four independent experiments.

We examined the effect of the TLR2 ligands PGN and Pam3CSK4 on the surface expression of FcεRI on LAD2 cells. Our results showed that comparing with the non-treated cells (grey line), pre-treatment of PGN or Pam3CSK4 for 24 h did not reduce the expression of FcεRI on LAD2 cells (black line) (Fig. 2A, B).

### TLR2 ligands inhibited anti-IgE induced intracellular calcium increase in LAD2 cells

**Figure 3:**
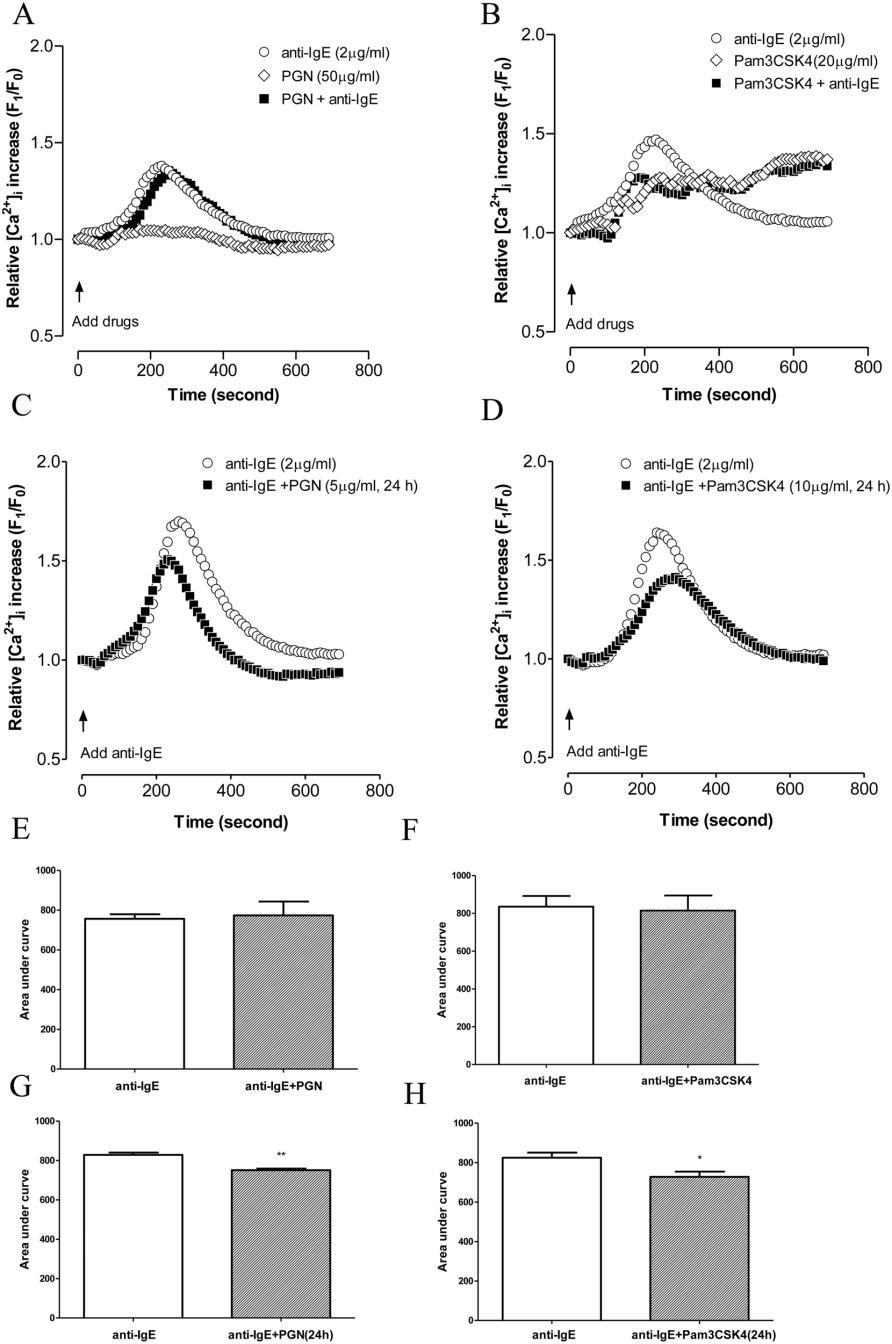
Effects of TLR2 ligands on anti-IgE induced calcium mobilization in LAD2 cells. (A, B) LAD2 cells were incubated with anti-IgE (○), PGN/Pam3CSK4 (◊), anti-IgE with PGN/Pam3CSK4 (▪), and calcium mobilization was measured at the same time. (C, D) Cells were incubated with PGN or Pam3CSK4 for 24 h prior to being challenged with anti-IgE (▪). Changes in [Ca^2+^]_i_ were compared in the presence or absent of PGN or Pam3CSK4. Error bars were omitted for the clarity of the graph. The peak levels of calcium influx were compared with area under the curves analysis (E, F, G, H). Significant differences following student *t*-test were indicated by asterisks: *p<0.05, **p<0.01 (n = 4–6).

Increase in cytosolic calcium is essential both for degranulation and for release of de novo synthesized mediators in mast cells [15]. Anti-IgE induced an increase of [Ca^2+^]_i_ with peak elevation at around 4 minutes after activation and gradually returned to normal level over the next 4 min. Pam3CSK4 induced a much lower but sustained level of [Ca^2+^]_i_ that was significant at around 3 min after activation while PGN did not cause intracellular calcium increase (Fig. 3A, B). Increase in calcium influx by Pam3CSK4 was not due to cytotoxicity as Pam3CSK4 did not influence cell viability of LAD2 cells as determined by WST-1 cell proliferation assay and trypan blue exclusion test following incubation of LAD2 cells with Pam3CSK4 for 30 min and 24 h (data not shown). When added simultaneously with anti-IgE, PGN did not influence anti-IgE induced calcium mobilization (Fig. 3A, E) while Pam3CSK4 altered the pattern of calcium increase induced by anti-IgE to that induced by Pam3CSK4 alone with reduced peak but sustained calcium influx (Fig. 3B). The AUC analysis thus did not show significant difference between the influx of calcium induced by anti-IgE alone and that induced by anti-IgE with Pam3CSK4 (Fig. 3F). Following 24 h pre-incubation, PGN and Pam3CSK4 both significantly inhibited the peak anti-IgE induced calcium increase (Fig. 3C, D) as indicated by the AUC analysis (Fig. 3G, H).

### Synergistic release of IL-8 induced by concomitant activation of LAD2 cells by anti-IgE and Pam3CSK4 was inhibited by blockers of calcineurin

**Figure 4:**
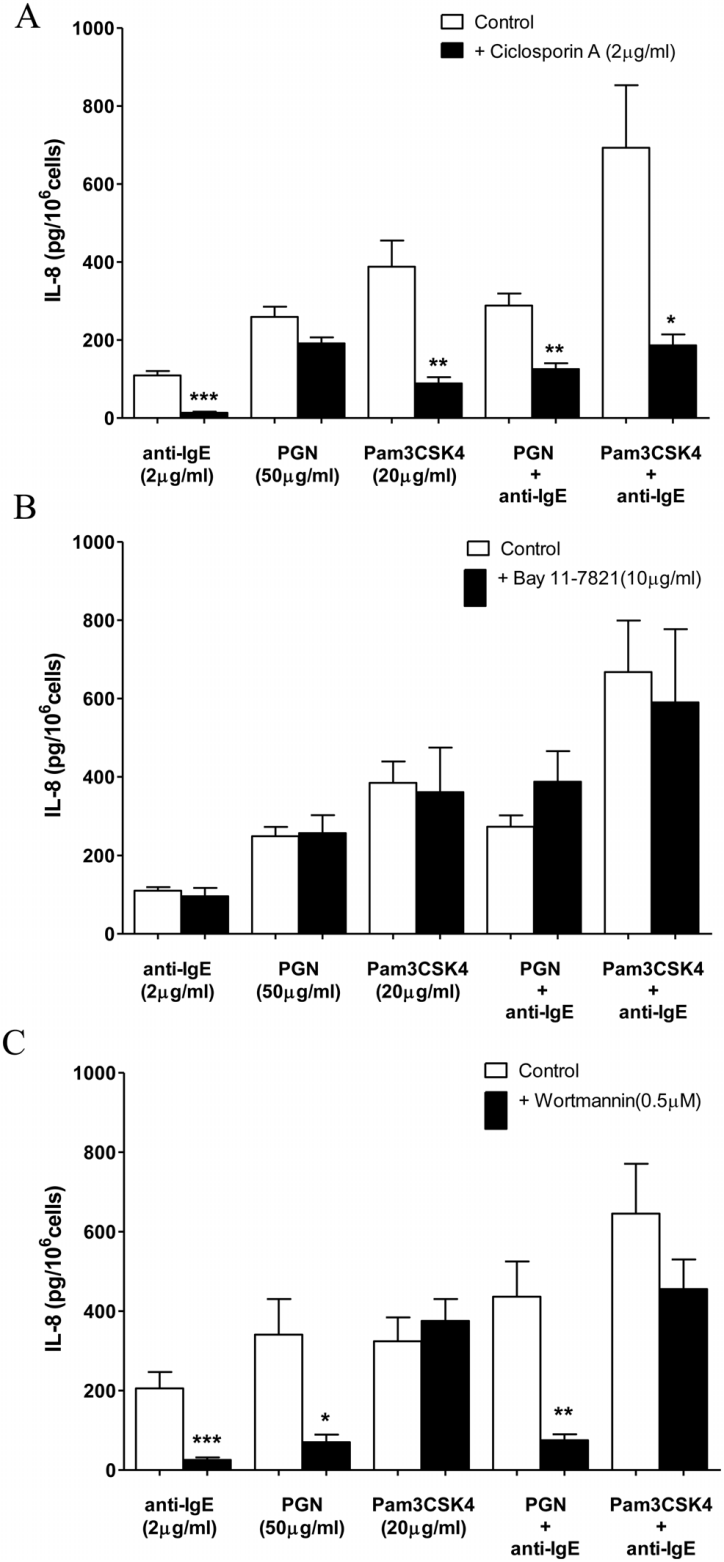
Effects of inhibitors of calcineurin, NF-κB and PI3K on TLR2 ligands and anti-IgE induced IL-8 release from LAD2 cells. (A) ciclosporin (2 µg/ml), (B) Bay 11-7821 (10 µM) or (C) wortmannin (0.5 µM) was incubated with LAD2 cells for 30 min before the addition of anti-IgE (2 µg/ml), PGN (50 µg/ml), Pam3CSK4 (20 µg/ml), anti-IgE with PGN or Pam3CSK4 for 24 h to induce the release of IL-8. The amounts of IL-8 release from activated LAD2 cells pre-incubated with an inhibitor and the corresponding control pre-incubated in culture medium were compared with student *t*-test. *p<0.05, **p<0.01, ***p<0.001 (n = 4–5).

In this series of experiments, LAD2 cells were pre-incubated with different enzyme inhibitors for 30 min before the addition of anti-IgE, PGN or Pam3CSK4. Activation of the phosphatase, calcineurin, induced by [Ca^2+^]_i_ increase contributes to the release of cytokines from anti-IgE stimulated mast cells [1]. As illustrated in Fig. 4A, IL-8 release induced by Pam3CSK4, anti-IgE and their combination were significantly blocked by the specific inhibitor of calcineurin, ciclosporin (CsA) (2 µg/ml). In contrast, CsA did not change PGN induced IL-8 release from LAD2 cells (Fig. 4A) but reduced the release of IL-8 induced by PGN together with anti-IgE by 50% (Fig. 4A).

NF-κB was shown to be involved in the release of cytokines and chemokines induced by agents that activate TLRs and FcεRI [11], [16]. However, the NF-κB inhibitor Bay 11-7821 (10 µM) failed to reduce IL-8 release from LAD2 cells incubated with PGN, Pam3CSK4, anti-IgE or the combinations of Anti-IgE with either of the two TLR2 ligands (Fig. 4B).

PI3K is well known to participate in human mast cells activity in term of degranulation and release of de novo synthesized mediators in response to various cell surface receptors including FcεRI [11], [12], [15]. Meanwhile, direct evidence for the requirement of PI3K in TLR2 signaling activation was showed by Arbibe et al [17]. The PI3K inhibitor wortmannin (0.5 µM) inhibited IL-8 release induced by PGN, anti-IgE and their combination (Fig. 4C). However, this inhibitor failed to demonstrate any effect on IL-8 release induced by Pam3CSK4 alone or in combination with anti-IgE (Fig. 4C).

### Involvement of MAPKs in TLR2 ligands and anti-IgE induced mast cell activation

**Figure 5:**
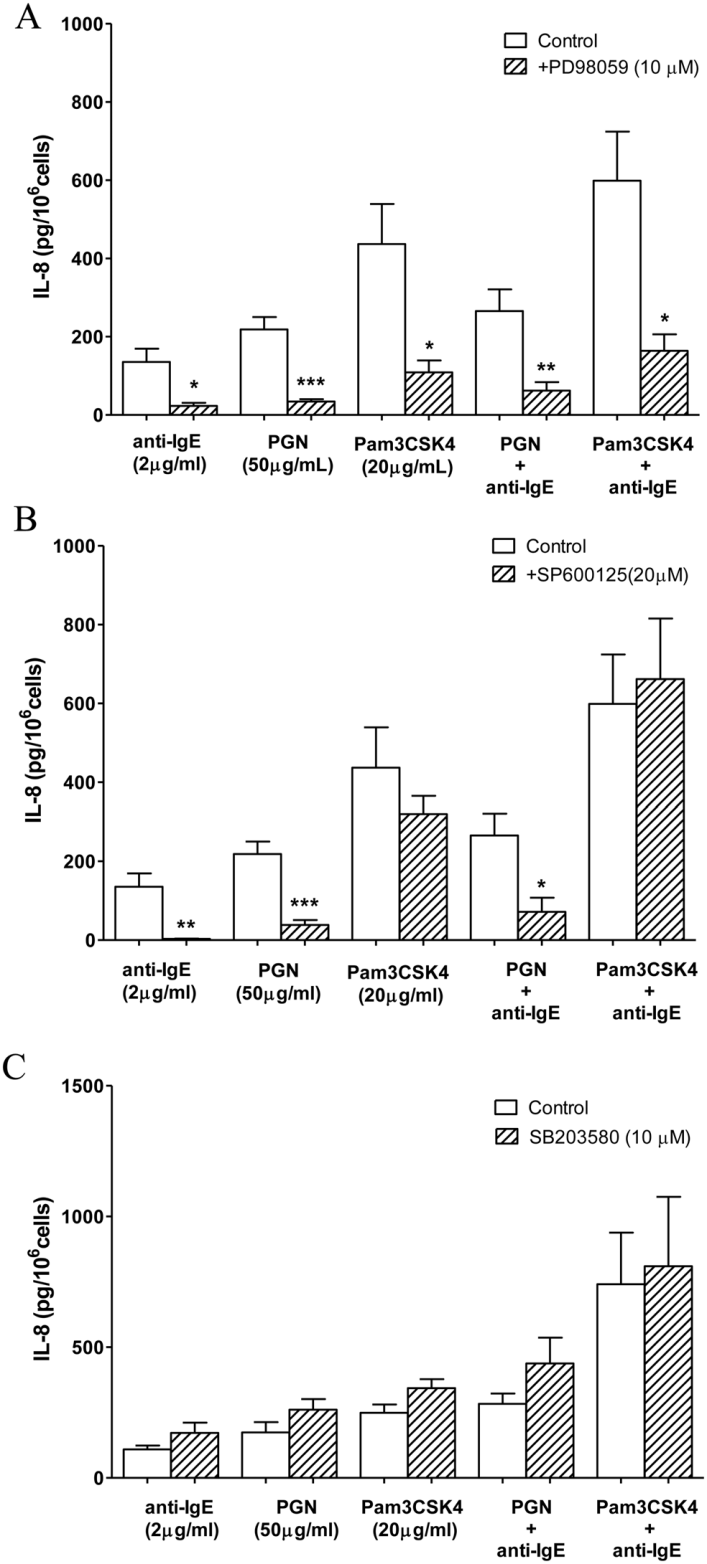
Effects of inhibitors of MAPKs on TLR2 ligands and anti-IgE induced IL-8 release from LAD2 cells. (A) ERK inhibitor, PD98059 (10 µM), (B) JNK inhibitor, SP600125 (20 µM) or (C) p38 inhibitor, SB2023580 (10 µM) was incubated with LAD2 cells for 30 min before the addition of anti-IgE (2 µg/ml), PGN (50 µg/ml), Pam3CSK4 (20 µg/ml), anti-IgE with PGN or Pam3CSK4 for 24 h to induce the release of IL-8. The amounts of IL-8 release from activated LAD2 cells pre-incubated with an inhibitor and the corresponding control pre-incubated in culture medium were compared with student *t*-test. *p<0.05, **p<0.01, ***p<0.01 (n = 4–6).

TLR2 ligands and anti-IgE both had been reported to induce the tyrosine phosphorylation of MAPKs, which are important signaling molecules for the production of wide variety of cytokines and chemokines in mast cells [3]. The ERK inhibitor PD98059 (10 µM) significantly inhibited IL-8 release from LAD2 cells induced separately by PGN, Pam3CSK4, anti-IgE and the combinations of anti-IgE with either TLR2 ligand (Fig. 5A). The JNK inhibitor SP600125 (20 µM) only inhibited PGN, anti-IgE and their combination induced release of IL-8 from LAD2 cells, but did not inhibit Pam3CSK4 induced IL-8 release, nor did it change the IL-8 release induced by Pam3CSK4 combined with anti-IgE (Fig. 5B). The p38 inhibitor SB203580 (10 µM) did not influence the release of IL-8 induced by PGN, Pam3CSK4, anti-IgE or the combination of anti-IgE with either of the TLR2 ligands (Fig. 5C).

## Discussion

Mast cells are pivotal immune effector cells not only in the context of allergic diseases, but also for the innate host defense system [18]–[20]. The discovery of the expression of Toll-like receptors on mast cells indicates that mast cells are capable of dealing with a great number of bacterial and viral infections [2]. Although clinical symptoms of asthma patients could be induced or worsened by microbial infections [21], there are also evidence showing that TLR2 ligands could reduce allergic airway inflammation in chronic respiratory sensitization to antigens, or inhibit Th2 response to mite allergen in human through decreasing the release of Th2 cytokines such as IL-5 and IL-13 [22], [23]. The contrasting observations may be due to the nature of the pathogenic molecules expressed by the microbes as implicated in the current study which demonstrated that TLR2/1 and TLR2/6 ligands could differentially modulate FcεRI mediated response in human mast cells. We showed that TLR2 ligands differentially modify human mast cell functions in response to antigen through inhibiting the degranulation and enhancing the release of *de* *novo* synthesized mediators.

Our observations that pre-incubation of LAD2 cells with PGN and Pam3CSK4 for 24 h suppressed anti-IgE induced degranulation in general agree with the inhibitory effect of TLR2 ligands on antigen induced degranulation reported in previous studies employing murine and human mast cells [1], [2], [8]. Yoshioka *et al* demonstrated that pre-incubation of LAD2 cells with PGN and lipoteichoic acid (LTA) for 48 h inhibited anti-IgE induced degranulation through down-regulation of FcεRI expression on LAD2 cells [2]. However, we could not observe any change in the expression of FcεRI in LAD2 cells pre-incubated with PGN or Pam3CSK4 for 24 h, while both TLR2 ligands could significantly inhibit anti-IgE induced degranulation from LAD2 cells. Furthermore, 24 h pre-incubation of PGN and Pam3CSK4 inhibited anti-IgE induced calcium mobilization in LAD2 cells, which was a necessary upstream signaling mechanism required for antigen induced mast cells degranulation [15]. In contrast to our previous study which observed that only Pam3CSK4 but not PGN could suppress calcium increase induced by the neuropeptide, substance P [24], the inhibitory action of PGN against anti-IgE induced calcium increase suggested that Pam3CSK4 and PGN modulate different sources of calcium. Since substance P induces mainly release of calcium from intracellular stores while FcεRI activation induces calcium influx through opening of cell membrane calcium channels, the activation of TLR2/6 heterodimer by PGN may thus suppress the functions of calcium channels. The calcium suppressing action of PGN was only apparent after 24 h incubation suggesting that the effect is not due to direct blocking of the calcium channels but related more to regulation of calcium channel expression or operation through generation of new regulatory molecules. In contrast, no pre-incubation was required for Pam3CSK4 to modify the calcium influx pattern induced by anti-IgE. Anti-IgE alone induced a sharp increase of calcium influx which returned quickly to the basal level, while Pam3CSK4 alone induced a lower level of calcium that is more sustained and this pattern is also observed in LAD2 cells incubated together with anti-IgE and Pam3CSK4. The low level of sustained calcium increase induced by Pam3CSK4 suggested that activation of the TLR2/1 heterodimer produced an immediate interference of the intracellular store of calcium but the low level of intracellular calcium release was not sufficient to induce the opening of cell surface calcium channels. Instead, the opening of cell surface calcium channels required for anti-IgE induced mast cell degranulation was suppressed probably due to inhibition or desensitization of the responses of intracellular calcium stores to FcεRI activation.

Previously, we have demonstrated that PGN and Pam3CSK4 induced the release of IL-8 by employing different signaling pathways in LAD2 cells. Furthermore, activation of different signaling pathways induced by PGN and Pam3CSK4 also resulted in opposite modulatory effect on LAD2 cells activation in response to substance P [24]. In the current study, Pam3CSK4 but not PGN acted in synergy with anti-IgE for IL-8 release from LAD2 cells which is the reverse of the modulatory actions of the two TLR2 agonists on substance P activated LAD2 cells. In addition to confirming that TLR2/1 ligand and TLR2/6 ligand induced different signaling in LAD2 cells, our results further illustrated that the mast cell activation mechanisms triggered by G-protein and IgE receptor are differently regulated by TLR2 agonists. Our observation did not provide evidence for crosstalk between TLR2/6 heterodimer and FcεRI activation mechanism in relation to IL-8 release since the combined IL-8 release are not different from the arithmetic sum of release individually induced by PGN and anti-IgE. In contrast, the releases of IL-8 induced by Pam3CSK4 alone and in combination with anti-IgE were blocked by ciclosporin, an inhibitor for Ca^2+^/calcineurin-mediated NFAT activation, and the ERK inhibitor PD98059. However, the PI3K inhibitor wortmannin and the JNK inhibitor SP600125 only inhibited IL-8 release induced by anti-IgE, but had no effect on that of Pam3CSK4 or the synergistic release induced by the combination of anti-IgE and Pam3CSK4. These observations indicate that crosstalk between FcεRI and TLR2/1 activation mechanisms converge at the level of calcineurin and ERK signaling pathways which in turn enhanced the effects of Pam3CSK4 in human mast cells as inhibition of FcεRI required signaling pathways had no effect on the synergistic release.

Although a number of signaling mechanisms, especially the MyD88 dependent pathways have been described for the activation of TLR2, there is a lack of literature which can clearly differentiate the detail signaling mechanisms induced by the TLR2/1 and TLR2/6 heterodimers. One of the possible upstream mechanisms would be the association of the TLR2/1 and TLR2/6 heterodimers with different lipid raft-resident proteins upon activation, which may result in the activation of different signaling pathways downstream. Triantafilou et al demonstrated that following activation by appropriate ligands, TLR2/TLR1 heterodimers were selectively recruited into lipid rafts which facilitated association with CD14, while TLR2/TLR6 were concentrated in lipid rafts which facilitated association with CD14 and CD36 [25]. One of the downstream signaling pathways that differentiates PGN and Pam3CSK4 induced IL-8 release is the calcineurin pathway as ciclosporin demonstrated no effect on IL-8 release induced by PGN but significant inhibition of those induced by Pam3CSK4 and the combination of anti-IgE and the TLR2/1 agonist. The conventional activation of calcineurin is initiated by an increase in intracellular calcium which was observed in LAD2 cells activated by anti-IgE and Pam3CSK4 as reported above. However, the amplitude of the calcium increase induced by the co-stimulation of Pam3CSK4 and anti-IgE was maintained at the same level as that induced by Pam3CSK4 alone which was lower than that induced by anti-IgE alone. This observation thus suggests that the ciclosporin sensitive synergistic release would be due to a calcium independent potentiation of calcineurin functions. Since the synergistic release of IL-8 was also blocked by the ERK inhibitor, PD98059, co-activation of LAD2 cells through TLR2/1 and FcεRI will most likely lead to synergistic activation of ERK as previously demonstrated in murine mast cells co-stimulated with TLR2 ligands and antigen [3]. Apart from inducing the *de* *novo* synthesis of IL-8 by directly initiating the gene transcription process, enhanced ERK activation may also improve the function of calcineurin by facilitating the dissociation of the endogenous calcineurin suppressor, RCAN1 (regulator of calcineurin 1), from the phosphatase as suggested by Shin et al and thus explains why the synergistic IL-8 release is also sensitive to the inhibitory action of ciclosporin [26].

TLR2 forms heterodimers with TLR1 and TLR6 to broaden the specificity of TLR-mediated pathogen recognition. TLR2/1 is activated by triacylated lipoproteins from different bacteria and lipoarabinomannan from mycobacteria, whereas TLR2/6 specifically recognizes diacyl lipopeptides from mycoplasma, zymosan from fungi, and lipoteichoic acid from group B streptococcus and staphylococcus [27]. Our current study has demonstrated that exposure of human mast cells to TLR2 activating microorganisms in general leads to suppression of the acute degranulation of mast cells mediated by FcεRI activation through mechanisms that reduce the level of intracellular calcium and thus reducing the contribution of mast cells to the acute phase of inflammation. On the contrary, the FcεRI induced release of cytokines such as IL8 is enhanced by microbes that activate TLR2/1 heterodimers but not those that activate TLR2/6 heterodimers. The current observations suggest that activation of TLR2 on mast cells may direct the functions of mast cells from being allergic responders towards being sentinel effectors in the host innate immune response. Together with our previous study on substance P [24], our current study further confirms that TLR2/1 and TLR2/6 heterodimers initiate different signaling pathways that produce differential actions on human mast cells stimulated through the peptidergic and FcεRI activation pathways. The contradicting clinical observations concerning the effects of microbial infections on allergic reactions may be due to the complex interactions between agonists of different TLR2 heterodimers with either allergens or mast cell activators of different mechanisms.

## Supporting Information

Figure S1 **Pam3CSK4 did not influence the proliferation of LAD2 cells.** LAD2 cells were plated at a concentration of 5×10^4^ cells per well in 96-well plates and cells were incubated with 20 µg/mL Pam3CSK4 (Invivogen) for 30 min or 24 h. 10 µL of WST-1 reagent (Beyotime, China) was added into each well and the absorbance was measured at 450 nm after 2 h of incubation using Model 680 Microplate Reader (Bio-Rad). Data are shown as mean ± SEM. Student *t*-test was employed to compare the significant difference between Pam3CSK4 treated and untreated cells. (n = 4).
(TIF)

## References

[1] Kasakura K, Takahashi K, Aizawa T, Hosono A, Kaminogawa S (2009) A TLR2 ligand suppresses allergic inflammatory reactions by acting directly on mast cells. Int Arch Allergy Immunol 150: 359–369. 19571568 10.1159/000226237

[2] Yoshioka M, Fukuishi N, Iriguchi S, Ohsaki K, Yamanobe H, et al (2007) Lipoteichoic acid downregulates FcepsilonRI expression on human mast cells through Toll-like receptor 2. J Allergy Clin Immunol 120: 452–461. 17481719 10.1016/j.jaci.2007.03.027

[3] Qiao H, Andrade MV, Lisboa FA, Morgan K, Beaven MA (2006) FcepsilonR1 and toll-like receptors mediate synergistic signals to markedly augment production of inflammatory cytokines in murine mast cells. Blood 107: 610–618. 16174756 10.1182/blood-2005-06-2271 PMC1895616

[4] Lauener RP, Birchler T, Adamski J, Braun-Fahrlander C, Bufe A, et al (2002) Expression of CD14 and Toll-like receptor 2 in farmers' and non-farmers' children. Lancet 360: 465–466. 12241724 10.1016/S0140-6736(02)09641-1

[5] Eder W, Klimecki W, Yu L, von Mutius E, Riedler J, et al (2004) Toll-like receptor 2 as a major gene for asthma in children of European farmers. J Allergy Clin Immunol 113: 482–488. 15007351 10.1016/j.jaci.2003.12.374

[6] Takenaka H, Ushio H, Niyonsaba F, Jayawardana ST, Hajime S, et al (2010) Synergistic augmentation of inflammatory cytokine productions from murine mast cells by monomeric IgE and toll-like receptor ligands. Biochem Biophys Res Commun 391: 471–476. 19914211 10.1016/j.bbrc.2009.11.082

[7] Saluja R, Delin I, Nilsson GP, Adner M (2012) FcepsilonR1-mediated mast cell reactivity is amplified through prolonged Toll-like receptor-ligand treatment. PLoS One 7: e43547. 22916277 10.1371/journal.pone.0043547 PMC3420882

[8] Kawahara T (2010) Inhibitory effect of heat-killed Lactobacillus strain on immunoglobulin E-mediated degranulation and late-phase immune reactions of mouse bone marrow-derived mast cells. Anim Sci J 81: 714–721. 21108693 10.1111/j.1740-0929.2010.00788.x

[9] Sandig H, Bulfone-Paus S (2012) TLR signaling in mast cells: common and unique features. Front Immunol 3: 185. 22783258 10.3389/fimmu.2012.00185 PMC3389341

[10] Zhang M, Murphy RF, Agrawal DK (2007) Decoding IgE Fc receptors. Immunol Res 37: 1–16. 17496343 10.1007/BF02686092

[11] Akira S, Takeda K (2004) Toll-like receptor signalling. Nat Rev Immunol 4: 499–511. 15229469 10.1038/nri1391

[12] Lin HY, Tang CH, Chen YH, Wei IH, Chen JH, et al (2010) Peptidoglycan enhances proinflammatory cytokine expression through the TLR2 receptor, MyD88, phosphatidylinositol 3-kinase/AKT and NF-kappaB pathways in BV-2 microglia. Int Immunopharmacol 10: 883–891. 20451669 10.1016/j.intimp.2010.04.026

[13] Askarian F, van Sorge NM, Sangvik M, Beasley FC, Henriksen JR, et al (2014) A Staphylococcus aureus TIR Domain Protein Virulence Factor Blocks TLR2-Mediated NF-kappaB Signaling. J Innate Immun 6: 485–498. 24481289 10.1159/000357618 PMC4198549

[14] Gwack Y, Feske S, Srikanth S, Hogan PG, Rao A (2007) Signalling to transcription: store-operated Ca2^+^ entry and NFAT activation in lymphocytes. Cell Calcium 42: 145–156. 17572487 10.1016/j.ceca.2007.03.007

[15] Gilfillan AM, Tkaczyk C (2006) Integrated signalling pathways for mast-cell activation. Nat Rev Immunol 6: 218–230. 16470226 10.1038/nri1782

[16] Saturnino SF, Prado RO, Cunha-Melo JR, Andrade MV (2010) Endotoxin tolerance and cross-tolerance in mast cells involves TLR4, TLR2 and FcepsilonR1 interactions and SOCS expression: perspectives on immunomodulation in infectious and allergic diseases. BMC Infect Dis 10: 240. 20707930 10.1186/1471-2334-10-240 PMC2930646

[17] Arbibe L, Mira JP, Teusch N, Kline L, Guha M, et al (2000) Toll-like receptor 2-mediated NF-kappa B activation requires a Rac1-dependent pathway. Nat Immunol 1: 533–540. 11101877 10.1038/82797

[18] McCall-Culbreath KD, Li Z, Zhang Z, Lu LX, Orear L, et al (2011) Selective, alpha2beta1 integrin-dependent secretion of il-6 by connective tissue mast cells. J Innate Immun 3: 459–470. 21502744 10.1159/000324832 PMC3186713

[19] Abel J, Goldmann O, Ziegler C, Holtje C, Smeltzer MS, et al (2011) Staphylococcus aureus evades the extracellular antimicrobial activity of mast cells by promoting its own uptake. J Innate Immun 3: 495–507. 21654154 10.1159/000327714 PMC11990236

[20] Sumbayev VV, Yasinska I, Oniku AE, Streatfield CL, Gibbs BF (2012) Involvement of hypoxia-inducible factor-1 in the inflammatory responses of human LAD2 mast cells and basophils. Plos One 7(3): e34259. 22470546 10.1371/journal.pone.0034259 PMC3314605

[21] Martin RJ (2006) Infections and asthma. Clin Chest Med 27: 87–98, vi. 10.1016/j.ccm.2005.10.004 16543054

[22] Fuchs B, Knothe S, Rochlitzer S, Nassimi M, Greweling M, et al (2010) A Toll-like receptor 2/6 agonist reduces allergic airway inflammation in chronic respiratory sensitisation to Timothy grass pollen antigens. Int Arch Allergy Immunol 152: 131–139. 20016195 10.1159/000265534

[23] Taylor RC, Richmond P, Upham JW (2006) Toll-like receptor 2 ligands inhibit TH2 responses to mite allergen. J Allergy Clin Immunol 117: 1148–1154. 16675345 10.1016/j.jaci.2006.02.014

[24] Yu YY, Yip KW, Tam IYS, Sam SW, NG CW, et al (2013) Differential effects of the Toll-like receptor 2 agonists, PGN and Pam3CSK4 on substance P induced human mast cell activation. Eur J Inflamm 3: 709–718. 10.1371/journal.pone.0112989 PMC4232580 25398056

[25] Triantafilou M, Gamper FG, Haston RM, Mouratis MA, Morath S, et al (2006) Membrane sorting of toll-like receptor (TLR)-2/6 and TLR2/1 heterodimers at the cell surface determines heterotypic associations with CD36 and intracellular targeting. J Biol Chem 281: 31002–31011. 16880211 10.1074/jbc.M602794200

[26] Shin SY, Yang HW, Kim JR, Heo WD, Cho KH (2011) A hidden incoherent switch regulates RCAN1 in the calcineurin-NFAT signaling network. J Cell Sci 124: 82–90. 21172821 10.1242/jcs.076034

[27] Ishii KJ, Coban C, Akira S (2005) Manifold mechanisms of Toll-like receptor-ligand recognition. J Clin Immunol 25: 511–521. 16380815 10.1007/s10875-005-7829-1

