# Supplementary figures and images for: Differential Effects of the Toll-Like Receptor 2 Agonists, PGN and Pam3CSK4 on Anti-IgE Induced Human Mast Cell Activation
Source: PLoS One. 2014 Nov 14;9(11):e112989. doi: 10.1371/journal.pone.0112989 (PMC4232580; doi:10.1371/journal.pone.0112989)

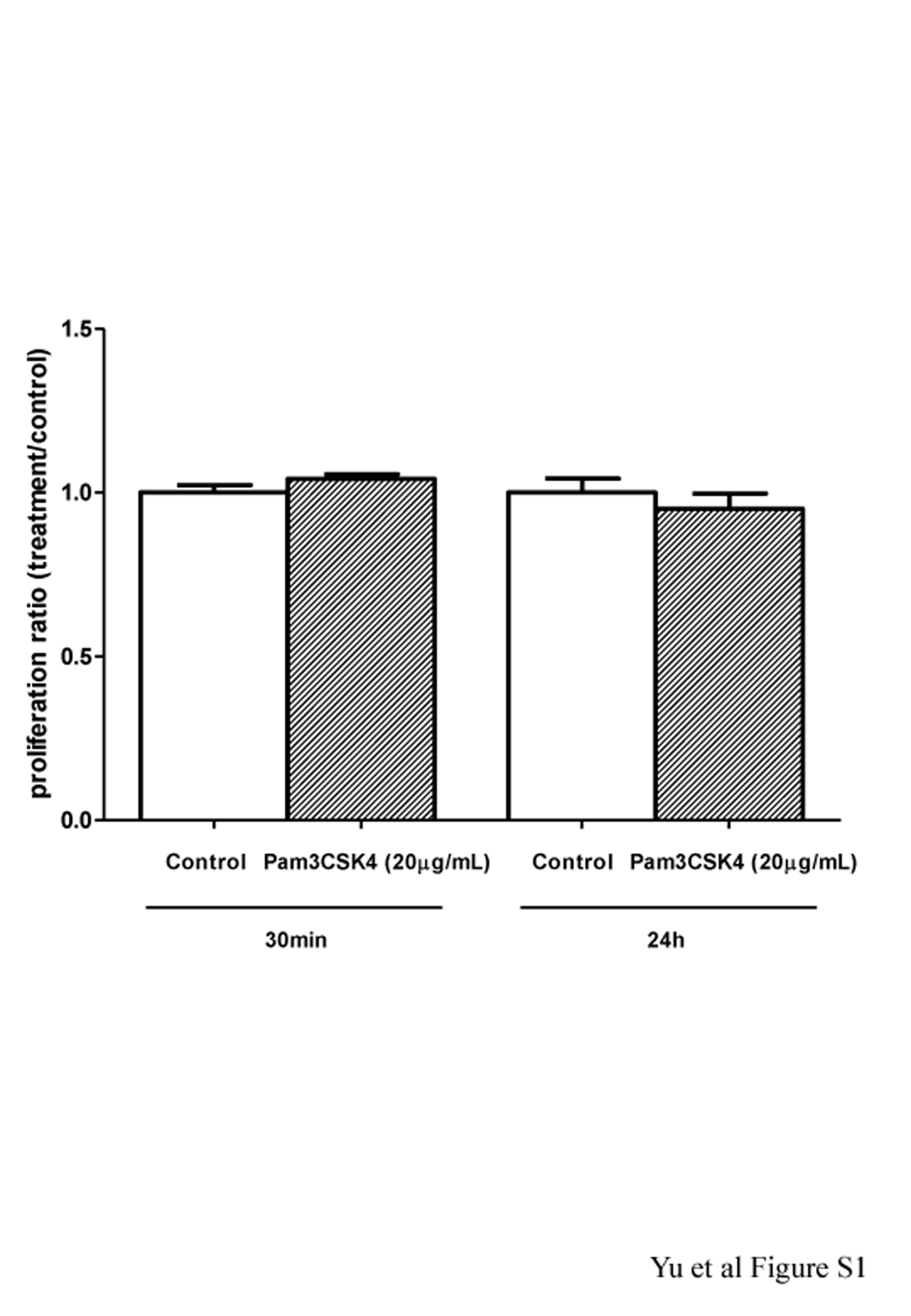

Supplement: Figure S1 — Pam3CSK4 did not influence the proliferation of LAD2 cells. LAD2 cells were plated at a concentration of 5×10^4^ cells per well in 96-well plates and cells were incubated with 20 µg/mL Pam3CSK4 (Invivogen) for 30 min or 24 h. 10 µL of WST-1 reagent (Beyotime, China) was added into each well and the absorbance was measured at 450 nm after 2 h of incubation using Model 680 Microplate Reader (Bio-Rad). Data are shown as mean ± SEM. Student t-test was employed to compare the significant difference between Pam3CSK4 treated and untreated cells. (n = 4). (TIF) [file pone.0112989.s001.tif]
